# Identification of the Active Constituents and Significant Pathways of Cangfu Daotan Decoction for the Treatment of PCOS Based on Network Pharmacology

**DOI:** 10.1155/2020/4086864

**Published:** 2020-02-21

**Authors:** Wenting Xu, Mengyu Tang, Jiahui Wang, Lihong Wang

**Affiliations:** Department of Reproduction, Zhangjiagang TCM Hospital Affiliated to Nanjing University of Chinese Medicine, Zhangjiagang, Suzhou, Jiangsu, China

## Abstract

**Background:**

Polycystic ovary syndrome (PCOS) is the most common female endocrine disease. Cangfu Daotan Decoction (CDD) can effectively relieve the clinical symptoms of PCOS patients.

**Methods:**

To explore the active ingredients and related pathways of CDD for treating PCOS, a network pharmacology-based analysis was carried out. The active ingredients of CDD and their potential targets were obtained from the TCM system pharmacology analysis platform. The obtained PCOS-related genes from OMIM and GeneCards were imported to establish protein-protein interaction networks in STRING. Finally, GO analysis and significant pathway analysis were conducted with the RStudio (Bioconductor) database.

**Results:**

A total of 111 active compounds were obtained from 1433 ingredients present in the CDD, related to 118 protein targets. In addition, 736 genes were found to be closely related to PCOS, of which 44 overlapped with CDD and were thus considered therapeutically relevant. Pathway enrichment analysis identified the AGE-RAGE signalling pathway in diabetic complications, endocrine resistance, the IL-17 signalling pathway, the prolactin signalling pathway, and the HIF-1 signalling pathway. Moreover, PI3K-Akt, insulin resistance, Toll-like receptor, MAPK, and AGE-RAGE were related to PCOS and treatment.

**Conclusions:**

CDD can effectively improve the symptoms of PCOS, and our network pharmacological analysis lays the foundation for future clinical research.

## 1. Background

Polycystic ovary syndrome (PCOS) is the most common endocrine disease in women and is characterized by abnormal adrenal and ovarian androgen secretion, ovulatory dysfunction, menstrual irregularity, acne [1], and polycystic ovarian morphological features, and in a significant proportion of patients, insulin resistance, with a prevalence between 5% and 15% [2, 3]. Although for a long period, studies on PCOS have focused on reproductive disorders [4], recent evidence suggests that PCOS is a heterogeneous disorder associated with a large number of severe metabolic implications as well as cardiovascular disease for affected women [5], which brings a heavy burden to the patient's family and to society. However, there is a generally poor understanding of its aetiology [6]. Genome-wide and molecular mechanism studies have identified certain candidate gene targets, although their role in the development of PCOS is still largely unknown; thus, early diagnosis, effective treatment, and the elucidation of underlying mechanisms are necessary [7, 8].

In the past few decades, oral contraceptive pills (OCPs) have been widely used in PCOS patients to regulate their menstrual cycles and reduce hyperandrogenism [4]. Clomiphene citrate and letrozole have both been used to induce ovulation in PCOS patients with fertility problems [9–11]. However, reports have been coming in regarding the side effects of OCPs in increasing the risk of venous thromboembolism (VTE) [12, 13]. Moreover, some experts have called attention to the unclear long-term risk-benefit ratio [11, 14, 15]. Due to the limitations of current treatments, traditional Chinese medicine (TCM) treatment for PCOS has become an important alternative therapy.

There is no specific Chinese medical term referring to “PCOS” in the ancient books of Chinese medicine. According to the clinical characteristics of PCOS, it is attributed to the category of irregular menstruation, infertility, obesity, etc. For thousands of years, TCM has been used to treat menstrual disorders, infertility, and obesity. There are a number of herbal formulas in treating the above diseases. A study showed that herbal medicine administration significantly relieved some of the symptoms of PCOS [16]. Specifically, the CDD consists of nine herbs: *Atractylodes lancea (Thunb.) Dc* (Cangzhu), *Cyperi Rhizoma* (Xiangfu), *Arisaematis Rhizoma* (Dannanxing), *Arum ternatum Thunb* (Banxia), *Zingiber officinale Roscoe* (Shengjiang), *Citrus reticulata* (Chenpi), *Aurantii Fructus* (Zhike), *Poria cocos* (*Schw.*) *Wolf* (Fuling), and Licorice (Gancao) and is one of the most common prescriptions for phlegm and dampness-type PCOS patients [17]. Another study showed that the CDD can significantly improve the pregnancy rate of infertile patients with PCOS, which might be associated with reduced insulin resistance, improved endometrial blood flow, and finally improved endometrial receptivity [18]. Although the CDD has been used clinically for gynaecological diseases for a long time, its mechanism of action is unclear because of its complex composition.

Therefore, it is necessary to clarify the biological basis and molecular nature of the TCM decoction. Network pharmacology has recently been developed as a new strategy and technique for elucidating complex pharmacological problems for new drug discovery [19]. In recent years, the TCM Systems Pharmacology (TCMSP) database and analysis platform has emerged as an ideal information convergence of the absorption, distribution, metabolism, and excretion (ADME) properties, drug-likeness, drug targets, associated diseases, and interaction networks of traditional medicines [20].

TCM decoctions have complex ingredients and multiple targets, and network pharmacology can predict novel compound targets and the potential pathways of action based on existing TCM decoctions and has helped clarify the mechanism of several TCM decoctions so far [21–23].

**Figure 1:**
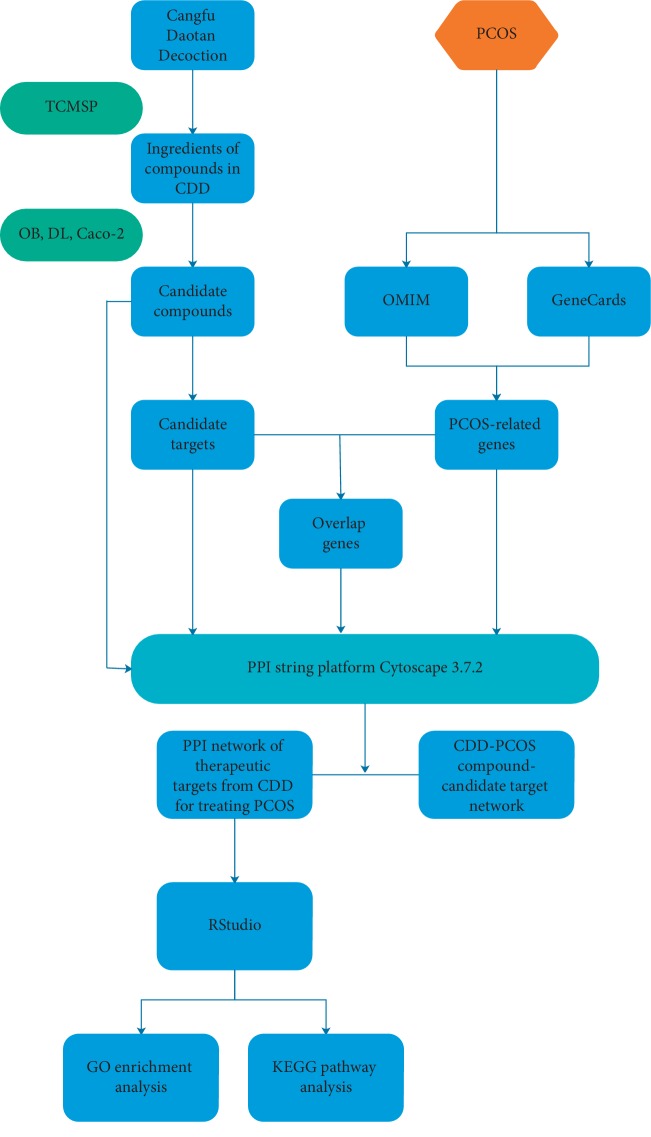
A schematic of the network pharmacology-based analysis of CDD for the treatment of PCOS.

In this study, we used the network pharmacology approach to explore the potential mechanism of action of CDD in treating PCOS. We first filtered the TCMSP database for active compounds of CDD and identified the targets, followed by mining for disease-related genes and network analysis of those genes (Figure 1).

## 2. Methods

### 2.1. Chemical Components of Each Herb in CDD

To screen the active ingredients of CDD, we used the TCMSP database (http://tcmspw.com/tcmsp.php) [20]. TCMSP is a unique systems pharmacology platform specifically designed for Chinese medicine that contains information on their ADME characteristics and captures the relationships between disease, targets, ingredients, and drugs [24].

### 2.2. Pharmacokinetic Prediction of CDD

CDD active ingredients were filtered mainly on the basis of oral bioavailability (OB), Caco-2 permeability (Caco-2), and drug-likeness (DL). OB, Caco-2, and DL are the three most key indicators of pharmacology. Specifically, the ingredients contained in the CDD meeting the criteria of OB ≥ 30%, Caco-2 ≥ 0.4, and DL ≥ 0.18 were chosen as candidate ingredients for further analysis.

OB refers to the percentage and rate of the release and absorption of active ingredients into the systemic blood circulation and is an important pharmacokinetic index of oral drugs [25]. It is also an important index to objectively evaluate the intrinsic quality of oral drugs [26], which is particularly important in the drug administration of most oral Chinese herbal formulas [27]. DL is defined as a complex balance of structural features and various molecular properties, which determine whether the particular molecule is similar to the known drugs [26]. These parameters, such as hydrogen bonding characteristics and hydrophobicity, mainly influence the behaviour of molecules in living organisms, which ultimately affects their transport properties, affinity for proteins, metabolic stability, and many other properties. Caco-2 permeability is widely used as a standard permeability screening assay for oral drug absorption related to drug permeability, which can predict the intestinal absorption of the ingredients and the fraction of the oral dose absorbed in humans [28].

In this study, OB ≥ 30%, DL ≥ 0.18, and Caco-2 ≥ 0.4 were regarded as a threshold for filtering possible candidate drugs.

### 2.3. Potential Targets of the Chemical Components of CDD

We chose the TCMSP database as the main source of component-target data and obtained the target protein names of each herb in CDD. Only the proteins that had interactions with the bioactive components in CDD we had already obtained were selected. Then, we converted the target protein names of the bioactive components of CDD into gene names with the species limited into “Homo sapiens” with the UniProt Knowledgebase (UniProtKB, http://www.uniprot.org).

### 2.4. Known Therapeutic Targets for PCOS

We collected PCOS targets from two sources. One is the GeneCards database (https://www.genecards.org/), which is a searchable, integrative database that predicts human genes. The knowledgebase automatically integrates gene-centric data from ∼150 web sources, including genomic, transcriptomic, proteomic, genetic, clinical, and functional information [29]. Another resource was the gene map in the Online Mendelian Inheritance in Man (OMIM) database [30] (https://omim.org/, updated on November 15, 2019), which is a comprehensive, authoritative, and timely knowledgebase of human genes and genetic disorders in the human genome. We searched the OMIM database with the keyword “PCOS.”

### 2.5. Protein-Protein Interactions

To illustrate the possible interaction between PCOS-related targets and the potential targets of CDD, we intersected the potential targets of CDD and PCOS-related drug targets and obtained the intersecting targets with RStudio 3.6.1 (Venn Diagram). The overlapping target proteins of PCOS and CDD were used to construct a protein-protein interaction (PPI) network with multiple protein patterns on the Search Tool for the Retrieval of Interacting Genes/Proteins (STRING) platform (https://string-db.org/, version 11.0). We set the organism type to “Homo sapiens” and left the default settings in place for the other parameters. Then, we exported the downloaded “string_interactions.tsv” file and imported it into Cytoscape 3.7.2 to obtain the PPI network and perform network analysis. In the network, nodes represent the target proteins, and edges represent the interaction between proteins.

### 2.6. GO and KEGG Pathway Enrichment Analysis

Gene Ontology (GO) and Kyoto Encyclopedia of Genes and Genomes (KEGG) pathway enrichment analysis are important methods used to describe the characteristics of candidate targets. We selected the standard *p* value cutoff of 0.05 and the q value of 0.05 and performed the enrichment analysis with RStudio 3.6.1 (Bioconductor, clusterProfiler).

## 3. Results

### 3.1. Composite Ingredients of CDD

A total of 1051 chemical ingredients of the nine herb medicines in CFDT were retrieved from TCMSP, including 49 ingredients in *Atractylodes lancea (Thunb.) Dc*, 104 ingredients in *Cyperi Rhizoma*, 123 ingredients in *Arisaematis Rhizoma*, 116 ingredients in *Arum ternatum Thunb*., 265 ingredients in *Zingiber officinale Roscoe*, 63 ingredients in *Citrus reticulata*, 17 ingredients in *Aurantii Fructus*, 34 ingredients in *Poria cocos* (*Schw*.) *Wolf*, and 280 ingredients in Licorice. 122 ingredients passed the filters of OB ≥ 30%, DL ≥ 0.18, and Caco-2 ≥ 0.4. The pharmacokinetic properties of the compounds are shown in Table 1.

### 3.2. Target Gene Prediction of CDD

**Figure 2:**
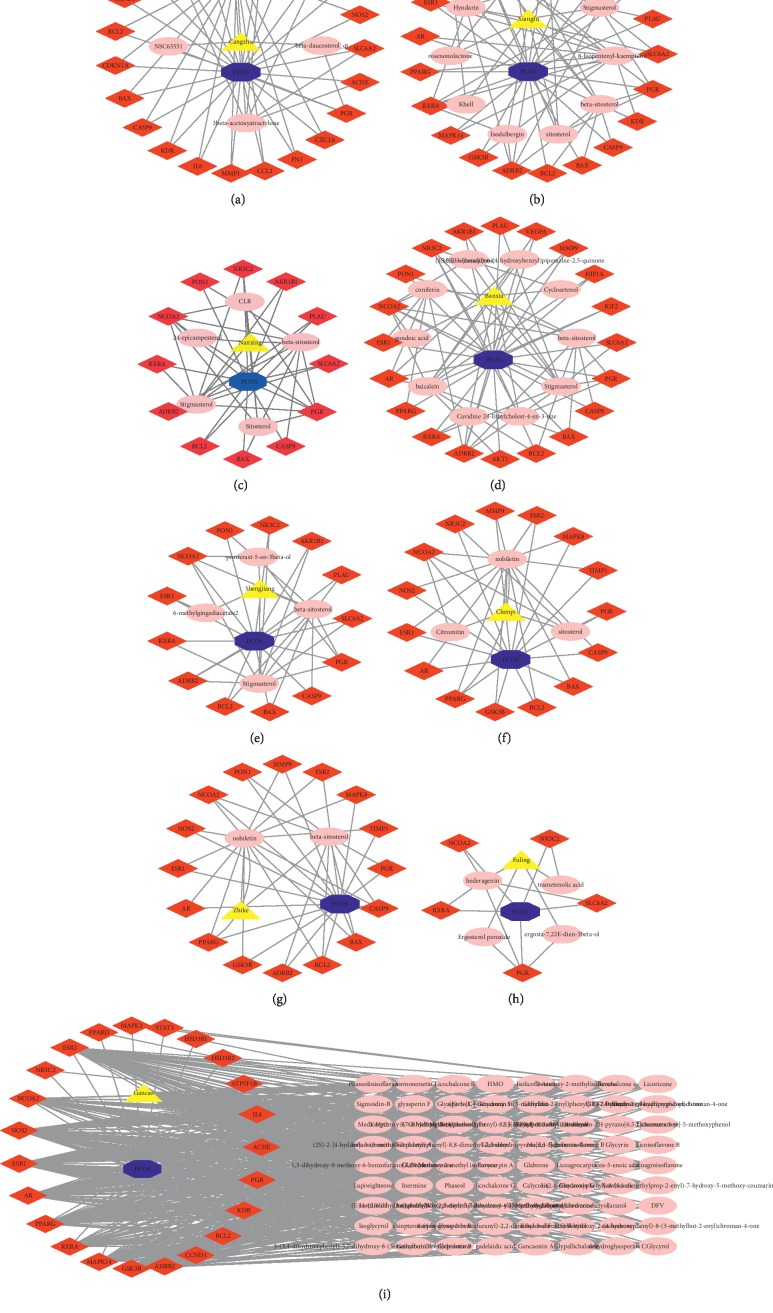
The potential targets from the ingredients of the herbs in CDD in the treatment of PCOS. The yellow triangle, red diamond, blue octagon, and the light pink ellipse nodes represent the herbs, targets, disease, and the molecules, respectively.

A total of 1433 potential targets from the 111 ingredients were retrieved from the TCMSP database (Figure 2). After eliminating the overlapping proteins, 118 related proteins were obtained and converted into gene names with the species limited into “Homo sapiens” based on the UniProtKB.

### 3.3. PCOS-Related Target Network

Research has shown that PCOS is a genetic predisposing, complex polygenic, multifactorial disorder [7, 31]. In this study, we obtained 736 targets related to PCOS through the GeneCards database (https://www.genecards.org/) and the OMIM database (https://omim.org/).

### 3.4. PPI Network Analysis

**Figure 3:**
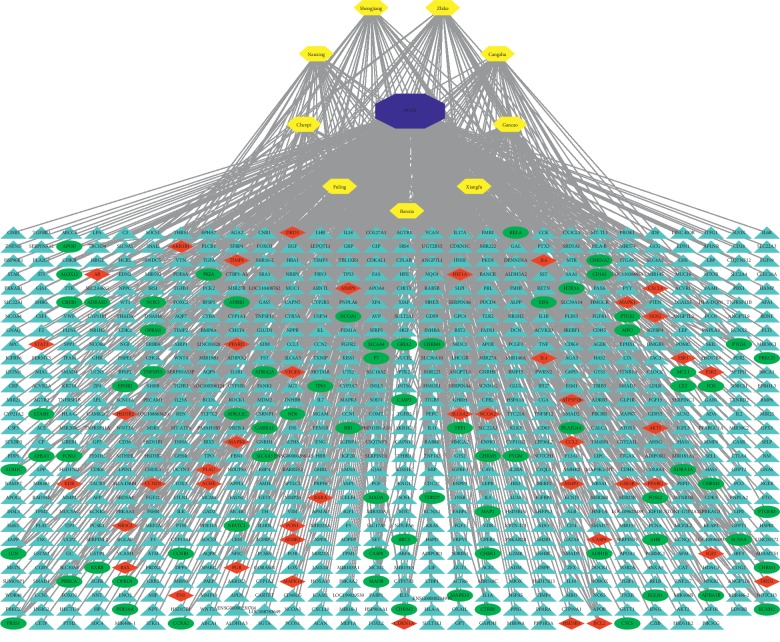
The CDD-PCOS-related drug targets. The blue octagon, yellow hexagon, green ellipse, mint green triangle, and red diamond represent disease, herbs, PCOS-related targets, CDD-related targets, and the common targets between CDD and PCOS, respectively.

**Figure 4:**
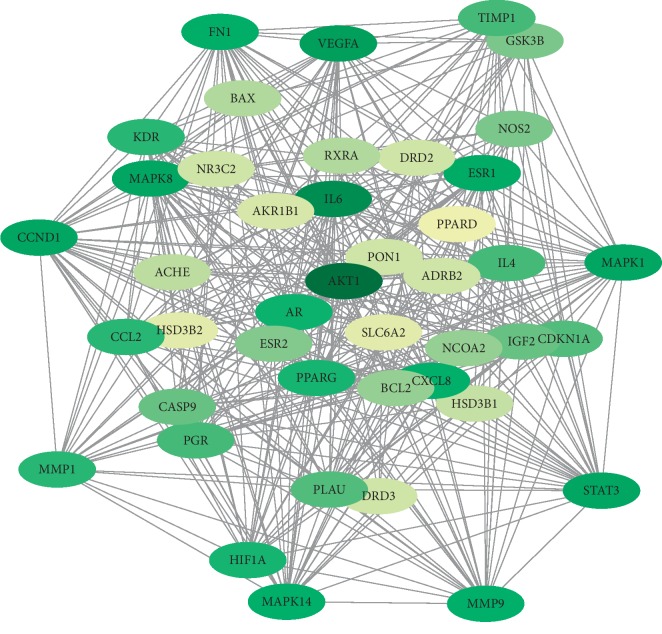
PPI network of targets for CDD in treating PCOS (from yellow to green, the degrees of freedom increase, and the thicker edges suggest stronger interactions).

Among the above 736 PCOS-related targets, CDD shared 44 common targets with PCOS (Figure 3). The 44 putative therapeutic targets were imported into the STRING database to establish the putative therapeutic target PPI network. The “string_interaction.tsv” file was then imported into Cytoscape 3.7.2 to perform network analysis. The network had 44 nodes, which interacted with 361 edges. The average node degree is 16.4, and the average local clustering coefficient is 0.698. From yellow to green, the degrees of freedom increase, and the thicker edges suggest stronger interactions (Figure 4). Our results indicated that the top mutual target proteins have various beneficial functions for treating PCOS at the molecular level.

In our network, the degree of the greenness of the nodes is high, and the numbers of edges of the nodes are 37 for AKT1; 32 for IL-6; 29 for VEGFA; 28 for CCND1, STAT3, MAPK1, and MAPK8; 27 for ESR1; 26 for FN1; 25 for MMP9, CXCL8, and MAPK14; 23 for AR; and 22 for PPARG and HIF1A. These results demonstrate that these targets are closely related to others in the network and consequently may play key roles in PCOS.

AKTs have been confirmed to play an important role in granulosa-lutein cell (GC) proliferation, and there is reciprocal feedback between AKT and androgen. This study showed that the high expression of AKT1 and AKT2 may cause GC dysfunction in PCOS patients through a possible relation with androgen [32]. Elevated serum levels of IL-6 in PCOS patients reflect low-grade chronic inflammation, which has been attributed to insulin resistance in PCOS [33]. In a PCOS rat model, increased expression of IL-6 and IL-11 was found to be associated with the AKT/STAT3 pathway. Increased levels of IL-6 and IL-11 stimulated adipocytes from adipose tissue of the PCOS rat model, which may activate AKT/STAT3 signalling and promote cell proliferation. Vascular endothelial growth factor (VEGF) plays an important role in the pathogenesis of many diseases. PCOS was also considered to be associated with high expression levels of VEGF. It was found that VEGF was a pivotal mediator of other factors to control ovary angiogenesis in women who underwent assisted reproductive technology (ART) procedures [34].

The MAPK signalling pathway is one of the most important signal transduction pathways in organisms and mediates physiological and pathological processes, such as the growth, development, and differentiation of organisms. Studies have shown that the MAPK signalling pathway plays an important role in the proliferation of ovarian granulosa cells [35, 36].

Research confirmed that PPARG rs709154 and ESR1 rs1999805 are significantly associated with PCOS risk in a Chinese population [37]. MMP9 may also be involved in the pathogenesis of PCOS [38]. HIF1A can induce glycolysis gene expression and promote anaerobic metabolism [39]. HIF-1a signalling was confirmed to be inhibited in a rat model with PCOS by increasing PHD activity [40].

### 3.5. Putative Therapeutic Compound-Putative Therapeutic Target Network Construction

**Figure 5:**
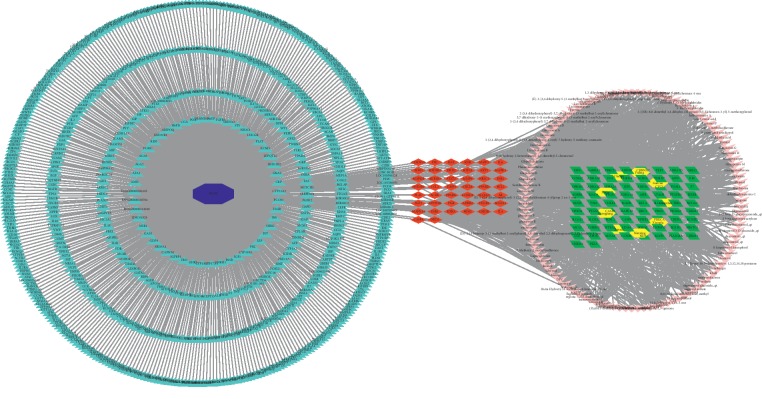
Herb-compound target-PCOS target network of CDD. The blue octagon, mint green triangle, yellow hexagon, green parallelogram, pink red V, and red diamond represent disease, PCOS-related targets, herbs, CDD-related targets, molecules, and the common targets, respectively.

The Merge function of Cytoscape 3.7.2 software was used to combine the Drug-Mol-Gene target network and the PCOS-Drug-Gene interaction network. The results showed that 111 compounds all together play a role in treating PCOS in the composite (Figure 5). The targets of 111 putative therapeutic targets in the treatment of PCOS include AKT1, IL-6, VEGFA, CCND1, STAT3, MAPK1, MAPK8, ESR1, FN1, MMP9, CXCL8, MAPK14, AR, PPARG, and HIF1A.

### 3.6. GO Functional Enrichment Analysis

**Figure 6:**
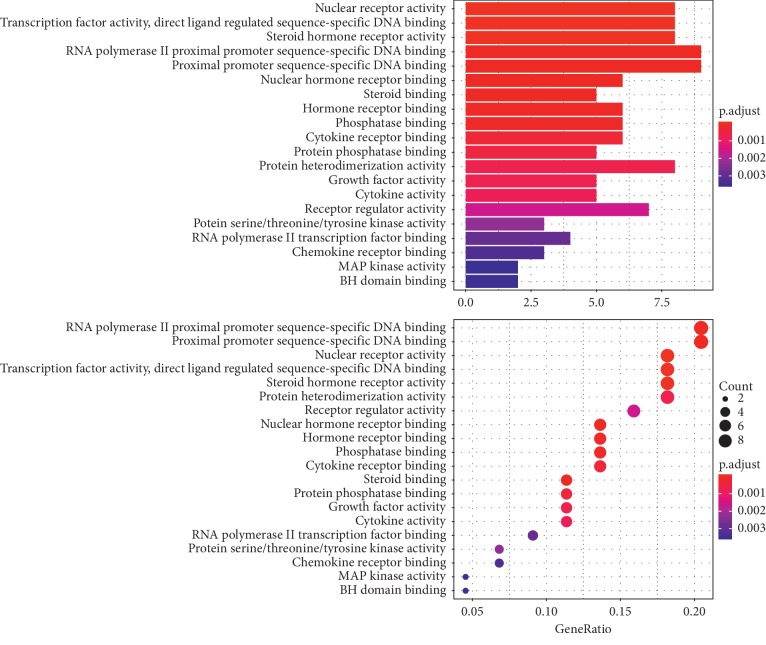
GO MF enrichment analysis of therapeutic targets of CDD in treating PCOS.

**Figure 7:**
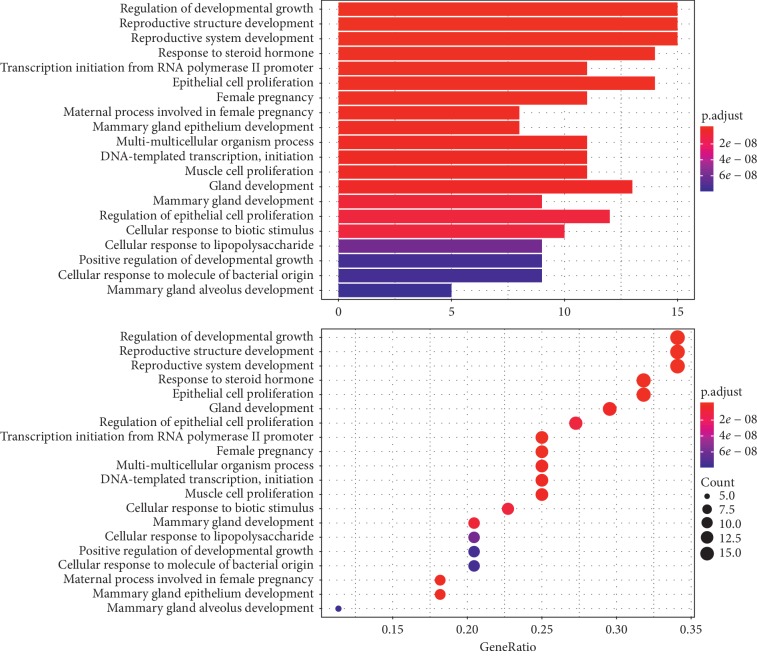
GO BP enrichment analysis of therapeutic targets of CDD in treating PCOS.

**Figure 8:**
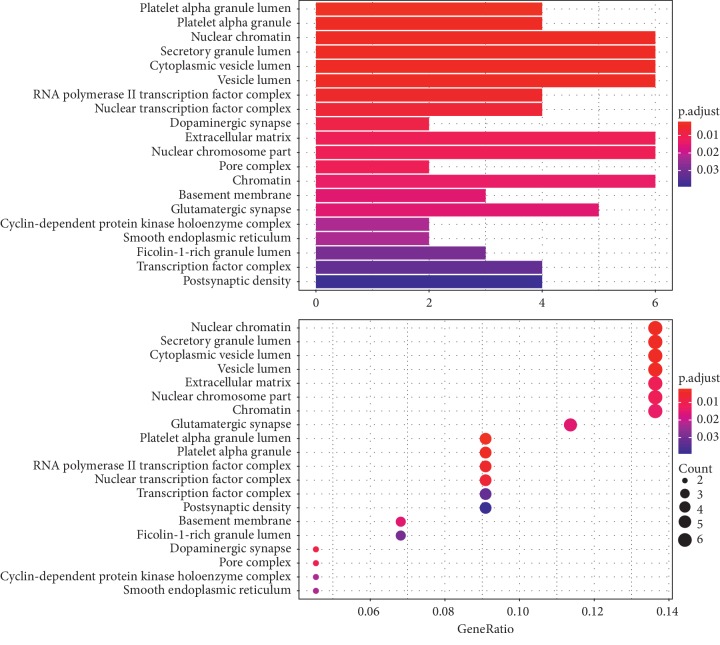
GO CC enrichment analysis of therapeutic targets of CDD in treating PCOS.

To further illuminate the biological effects involved in the treatment of PCOS with CDD, we performed GO analysis of the 44 PCOS-related putative potential therapeutic target genes. GO annotation and enrichment of the genes encoding CDD were conducted from three aspects: molecular function (MF), biological process (BP), and cellular composition (CC). The most enriched terms in GO analysis are shown in Figures 6–8.

In detail, the top 18 terms in the GO BP category were mainly enriched in the regulation of developmental growth, reproductive system development, response to steroid hormone, epithelial cell proliferation, gland development, regulation of epithelial cell proliferation, transcription initiation from RNA polymerase II promoter, female pregnancy, multi-multicellular organism process, DNA-templated transcription, initiation, muscle cell proliferation, ossification, regulation of DNA-binding transcription factor activity, response to radiation, ameboidal-type cell migration, autophagy, and process utilizing autophagic mechanism (Figure 6).

The top 19 terms in the GO CC category were mainly enriched in nuclear chromatin, secretory granule lumen, cytoplasmic vesicle lumen, vesicle lumen, extracellular matrix, nuclear chromosome part, chromatin, glutamatergic synapse, platelet alpha granule lumen, platelet alpha granule, RNA polymerase II transcription factor complex, nuclear transcription factor complex, transcription factor complex, postsynaptic density, receptor complex, asymmetric synapse, neuron to neuron synapse, postsynaptic specialization, and basement membrane (Figure 7).

The top 19 terms in the GO MF category were mainly enriched in RNA polymerase II proximal promoter sequence-specific DNA binding, proximal promoter sequence-specific DNA binding, nuclear receptor activity, transcription factor activity, direct ligand regulated sequence-specific DNA binding, steroid hormone receptor activity, protein heterodimerization activity, receptor regulator activity, nuclear hormone receptor binding, hormone receptor binding, phosphatase binding, cytokine receptor binding, receptor ligand activity, chromatin binding, steroid binding, protein phosphatase binding, growth factor activity, cytokine activity, protein serine/threonine kinase activity, DNA-binding transcription activator activity, RNA polymerase II-specific, transcription coregulator activity, and RNA polymerase II transcription factor binding (Figure 8).

### 3.7. Pathway Analysis

To clarify the biological actions of these targets, we performed KEGG analysis and pathway enrichment analysis with the RStudio (Bioconductor) database. We listed the genes of major PCOS putative therapeutic targets and imported them into RStudio to generate relevant pathways that might have an important influence on the biological process of CDD in treating PCOS. Pathways with an adjusted *p* value < 0.05 were considered significant.

**Figure 9:**
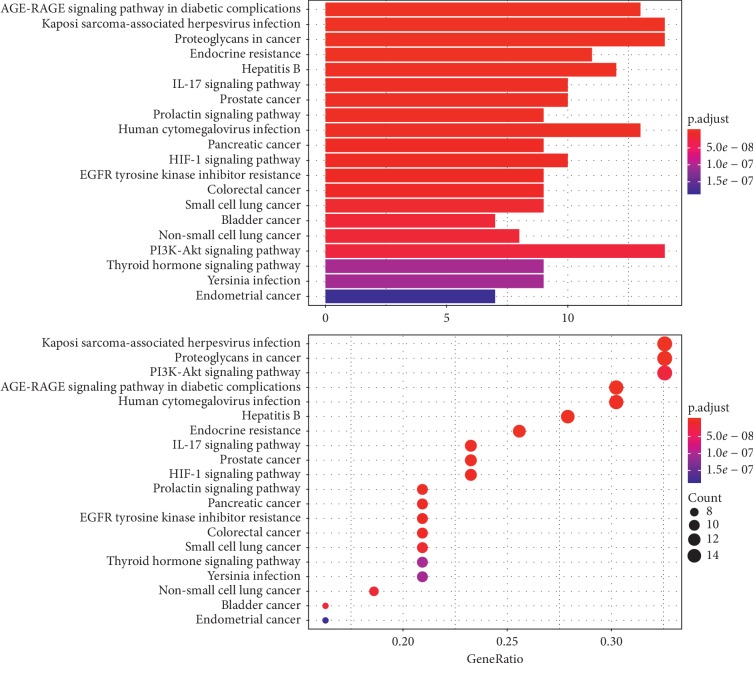
KEGG enrichment pathway analysis of therapeutic targets of CDD in treating PCOS.

A total of 123 signalling pathways were significantly enriched through pathway enrichment analysis. In the histogram and bubble diagram (Figure 9), the colour, size, and length of the nodes and bars were determined according to the numbers and *p* values of the related genes. The colour from blue to red reflects the adjusted *p* values from large to small, and the node size and bar length indicate the number of related genes. The histogram and bubble chart were separately arranged according to the *p* value and the number of related genes. According to the *p* value, the AGE-RAGE signalling pathway in diabetic complications, Kaposi sarcoma-associated herpesvirus infection, proteoglycans in cancer, endocrine resistance, hepatitis B, the IL-17 signalling pathway, prostate cancer, the prolactin signalling pathway, human cytomegalovirus infection, pancreatic cancer, the HIF-1 signalling pathway, and EGFR tyrosine kinase inhibitor resistance were within the top 12 terms. According to the number of related genes, Kaposi sarcoma-associated herpesvirus infection, proteoglycans in cancer, the PI3K-Akt signalling pathway, the AGE-RAGE signalling pathway in diabetic complications, human cytomegalovirus infection, hepatitis B, endocrine resistance, the IL-17 signalling pathway, prostate cancer, the HIF-1 signalling pathway, Epstein–Barr virus infection, and the prolactin signalling pathway were in the top 12 terms. The overlapping terms of the above lists included the AGE-RAGE signalling pathway in diabetic complications, endocrine resistance, the IL-17 signalling pathway, the prolactin signalling pathway, and the HIF-1 signalling pathway.

AGE-RAGE signalling may activate multiple intracellular signalling pathways involving protein kinase C and MAPKs and may influence NF-κB activity. Research has shown an association between PCOS and chronic inflammatory factors, proinflammatory cytokines such as interleukin-1, interleukin-6, interleukin-17 [41–43], and tumour necrosis-*α*, and a variety of inflammation-related genes, including VEGF, tissue factor, and RAGE [44, 45].

Prolactin (PRL) is involved in a variety of biological functions, including osmotic regulation, reproduction, lactation, endocrinology and metabolism, growth and development, and immunomodulation. It is a polypeptide hormone secreted by the pituitary gland that regulates the female reproductive and endocrine system by activating pathways, including the MAPK and PI3K pathways [46]. Insulin resistance is a common type of endocrine resistance and is related to PCOS by deregulating the IRS-PI3K-Akt signalling axis that integrates aberrant inflammatory responses [47].

A previous study also revealed that hypoxia-inducible factor-1a (HIF-1a) mediated endothelin-2 (ET-2) signalling and plays an important role in ovulation in rats and indicated that HIF-1a signalling is inhibited in a PCOS rat model by increasing PHD activity [40].

## 4. Discussion

TCM plays a crucial role in the pharmaceutical industry, alternative medicine, and many other fields [48–50]. However, due to the complex chemical composition and unclear pharmacological mechanism of action of TCMs, it faces great obstacles in pharmacological research, quality control and supervision, modernization, and internationalization [51].

TCM gives priority to the concept of holism in diagnosis and pays more attention to the overall changes in function. The treatment is based on syndrome differentiation, emphasizing the recovery of overall function, which is similar to the network concept of network pharmacology [52].

Traditional research methods on TCM mainly rely on pharmacochemical and pharmacological experiments, which have many problems, such as low data flux, low precision, long cycles, and high costs. The construction and development of a network pharmacology analysis platform, TCM database, and target prediction technology has brought new ideas and strategies for the study on the basis and mechanism of the pharmacodynamic substances of TCM to clarify characteristics such as the good curative effects, high safety profile, and multicomponents and multitargets of TCM [53]. These developments also lead to a new direction for the modernization and internationalization of TCM [54].

The combination of bioinformatics, computer technology, and experiments in the study of the pharmacodynamic components and potential targets of TCM broke through the deficiency of traditional studies is more rapid, flexible, and accurate and has been widely used in research on the network pharmacology of TCM [55].

TCM has a long history in treating menstrual diseases and infertility related to PCOS and has achieved good effects [56]. In this study, several network pharmacology-based methods were used to predict potential targets. This approach provides new clues to exploring ethnopharmacology and herbal or even TCM formulas. Through the analysis of the putative therapeutic target network and biological functions in this study, the potential pharmacological and molecular mechanisms of CDD in treating PCOS were preliminary revealed.

In this study, the preliminary analysis based on network pharmacology provided a basis for subsequent studies on the pharmacodynamic ingredients and mechanisms of CDD. However, due to the limitations of the screening conditions and the TCM database, this study also has some shortcomings. Although a large number of targets and pathways were obtained through network pharmacology screening, these results must be verified by subsequent pharmacological experiments. In this study, the active components of CDD and their molecular mechanisms in PCOS were predicted overall, and a variety of potential therapeutic targets were explored. Thus, this study lays a good theoretical foundation for the next step of experimental verification and provides directions for further research on the molecular mechanism of PCOS.

## Figures and Tables

**Table 1:** A list of the active compounds in CDD.

Mol ID	Molecule name	0B (%)	Caco-2	DL
MOL001484	Inermine	75.18	0.89	0.54
MOL001792	DFV	32.76	0.51	0.51
MOL000211	Mairin	55.38	0.73	0.78
MOL002311	Glycyrol	90.78	0.71	0.67
MOL000239	Jaranol	50.83	0.61	0.29
MOL002565	Medicarpin	49.22	1	0.34
MOL000359	Sitosterol	36.91	1.32	0.75
MOL003656	Lupiwighteone	51.64	0.68	0.37
MOL003896	7-Methoxy-2-methyl isoflavone	42.56	1.16	0.2
MOL000392	Formononetin	69.67	0.78	0.21
MOL000417	Calycosin	47.75	0.52	0.25
MOL004805	(2S)-2-[4-Hydroxy-3-(3-methylbut-2-enyl)phenyl]-8,8-dimethyl-2,3-dihydropyrano[2,3-f]chromen-4-one	31.79	1	0.72
MOL004806	Euchrenone	30.29	1.09	0.57
MOL004808	Glyasperin B	65.22	0.47	0.44
MOL004810	Glyasperin F	75.84	0.43	0.54
MOL004811	Glyasperin C	45.56	0.71	0.4
MOL004814	Isotrifoliol	31.94	0.53	0.42
MOL004815	(E)-1-(2,4-Dihydroxyphenyl)-3-(2,2-dimethylchromen-6-yl)prop-2-en-l-one	39.62	0.66	0.35
MOL004820	Kanzonol W	50.48	0.63	0.52
MOL004827	Semilicoisoflavone B	48.78	0.45	0.55
MOL004828	Glepidotin A	44.72	0.79	0.35
MOL004829	Glepidotin B	64.46	0.46	0.34
MOL004833	Phaseolinisoflavan	32.01	1.01	0.45
MOL004835	Glypallichalcone	61.6	0.76	0.19
MOL004838	8-(6-Hydroxy-2-benzofuranyl)-2,2-dimethyl-5-chromenol	58.44	1	0.38
MOL004841	Licochalcone B	76.76	0.47	0.19
MOL004848	Licochalcone G	49.25	0.64	0.32
MOL004849	3-(2,4-Dihydroxyphenyl)-8-(1,1-dimethylprop-2-enyl)-7-hydroxy-5-methoxy-coumarin	59.62	0.4	0.43
MOL004855	Licoricone	63.58	0.53	0.47
MOL004856	Gancaconin A	51.08	0.8	0.4
MOL004857	Gancaconin B	48.79	0.58	0.45
MOL004863	3-(3,4-Dihydroxyphenyl)-5,7-dihydroxy-8-(3-methylbut-2-enyl)chromone	66.37	0.52	0.41
MOL004864	5,7-Dihydroxy-3-(4-methoxyphenyl)-8-(3-methylbut-2-enyl)chromone	30.49	0.9	0.41
MOL004866	2-(3,4-Dihydroxyphenyl)-5,7-dihydroxy-6-(3-methylbut-2-enyl)chromone	44.15	0.48	0.41
MOL004879	Glycyrin	52.61	0.59	0.47
MOL004882	Licocoumarone	33.21	0.84	0.36
MOL004884	Licoisoflavone B	38.93	0.46	0.55
MOL004891	Shinpterocarpin	80.3	1.1	0.73
MOL004898	(E)-3-[3,4-Dihydroxy-5-(3-methylbut-2-enyl)phenyl]-1-(2,4-dihydroxyphenyl)prop-2-en-l-one	46.27	0.41	0.31
MOL004910	Glabridin	53.25	0.97	0.47
MOL004911	Glabridin	52.9	0.97	0.31
MOL004912	Glabrone	52.51	0.59	0.5
MOL004913	1,3-Dihydroxy-9-methoxy-6-benzofurano[3,2-c]chromone	48.14	0.48	0.43
MOL004915	Eurycarpin A	43.28	0.43	0.37
MOL004935	Sigmoidin B	34.88	0.42	0.41
MOL004941	(2R)-7-Hydroxy-2-(4-hydroxyphenyl)chroman-4-one	71.12	0.41	0.18
MOL004945	(2S)-7-Hydroxy-2-(4-hydroxyphenyl)-8-(3-methylbut-2-enyl)chromen-4-one	36.57	0.72	0.32
MOL004948	Isoglycyrol	44.7	0.91	0.84
MOL004949	Isolicoflavonol	45.17	0.54	0.42
MOL004957	HMO	38.37	0.79	0.21
MOL004959	1-Methoxyphaseollidin	69.98	1.01	0.64
MOL004966	3′-Hydroxy-4′-0-methylglabridin	43.71	1	0.57
MOL000497	Licochalcone A	40.79	0.82	0.29
MOL004974	3′-Methylglabridin	46.16	0.94	0.57
MOL004978	2-[(3R)-8,8-Dimethyl-3,4-dihydro-2H-pyrano[6,5-f]chromen-3-yl]-5-methoxyphenol	36.21	1.12	0.52
MOL004980	Inflacoumarin A	39.71	0.73	0.33
MOL004985	Icso-5-enoic acid	30.7	1.22	0.2
MOL004988	Kanzonol F	32.47	1.18	0.89
MOL004991	7-Acetoxy-2-methylisoflavone	38.92	0.74	0.26
MOL004993	8-Prenylated eriodictyol	53.79	0.43	0.4
MOL004996	Gadelaidic acid	30.7	1.2	0.2
MOL000500	Vestitol	74.66	0.86	0.21
MOL005000	Gancaonin G	60.44	0.78	0.39
MOL005001	Gancaonin H	50.1	0.6	0.78
MOL005003	Licoagrocarpin	58.81	1.23	0.58
MOL005007	Glyasperin M	72.67	0.49	0.59
MOL005012	Licoagroiosoflavone	57.28	0.71	0.49
MOL005016	Odoratin	49.95	0.42	0.3
MOL005017	Phaseol	78.77	0.76	0.58
MOL005018	Xambioona	54.85	1.09	0.87
MOL005020	Dehydroglyasperin C	53.82	0.68	0.37
MOL000173	Wogonin	30.68	0.79	0.23
MOL000184	NSC63551	30.25	1.42	0.76
MOL000186	Stigmasterol 3-0-beta-D-glucopyranoside_qt	43.83	1.31	0.76
MOL000188	3*β*-Acetoxyatractylone	40.57	1.22	0.22
MOL000085	Beta-daucosterol_qt	36.91	1.3	0.75
MOL000088	Beta-sitosterol 3-0-glucoside_qt	36.91	1.3	0.75
MOL000092	Daucosterin_qt	36.91	1.42	0.76
MOL000094	Daucosterol_qt	36.91	1.3	0.76
MOL003542	8-Isopentenyl-kaempferol	38.04	0.53	0.39
MOL000358	Beta-sitosterol	36.91	1.32	0.75
MOL004027	1,4-Epoxy-16-hydroxypheneicos-1,3,12,14,18-pentaene	45.1	1.28	0.24
MOL004053	Isodalbergin	35.45	0.8	0.2
MOL004058	Khell	33.19	1.12	0.19
MOL004068	Rosenonolactone	79.84	0.72	0.37
MOL004071	Hyndarin	73.94	1	0.64
MOL004074	Stigmasterol glucoside_qt	43.83	1.31	0.76
MOL004077	Sugeonyl acetate	45.08	0.72	0.2
MOL000449	Stigmasterol	43.83	1.44	0.76
MOL013146	8,11,14-Docosatrienoic acid, methyl ester	43.23	1.53	0.3
MOL001510	24-Epicampesterol	37.58	1.43	0.71
MOL000953	CLR	37.87	1.43	0.68
MOL001755	24-Ethylcholest-4-en-3-one	36.08	1.46	0.76
MOL002670	Cavidine	35.64	1.08	0.81
MOL002714	Baicalein	33.52	0.63	0.21
MOL005030	Gondoic acid	30.7	1.2	0.2
MOL000519	Coniferin	31.11	0.42	0.32
MOL006936	10,13-Eicosadienoic	39.99	1.22	0.2
MOL006957	(3S,6S)-3-(Benzyl)-6-(4-hydroxybenzyl)piperazine-2,5-quinone	46.89	0.41	0.27
MOL003578	Cycloartenol	38.69	1.53	0.78
MOL006129	6-Methylgingediacetate2	48.73	0.55	0.32
MOL001771	Poriferast-5-en-3beta-ol	36.91	1.45	0.75
MOL008698	Dihydrocapsaicin	47.07	0.98	0.19
MOL005815	Citromitin	86.9	0.88	0.51
MOL005828	Nobiletin	61.67	1.05	0.52
MOL000275	Trametenolic acid	38.71	0.52	0.8
MOL000282	Ergosta-7,22E-dien-3beta-ol	43.51	1.32	0.72
MOL000283	Ergosterol peroxide	40.36	0.84	0.81
MOL000287	3beta-Hydroxy-24-methylene-8-lanostene-21-oic acid	38.7	0.61	0.81
MOL000296	Hederagenin	36.91	1.32	0.75

## Data Availability

The data used to support the findings of this study are available from the corresponding author upon request.
